# Cardiovascular Involvement in Connective Tissue Disease: The Role of Interstitial Lung Disease

**DOI:** 10.1371/journal.pone.0121976

**Published:** 2015-03-16

**Authors:** XiaoBing Wang, MeiNa Lou, Yongji Li, WenJing Ye, ZhiYong Zhang, Xiufen Jia, HongYing Shi, XiaoChun Zhu, LiangXing Wang

**Affiliations:** 1 Department of Rheumalogy, The First Affiliated Hospital of Wenzhou Medical University, Zhejiang, China; 2 Department of Cardiology, The First Affiliated Hospital of Wenzhou Medical University, Zhejiang, China; 3 Radiology Department, The First Affiliated Hospital of Wenzhou Medical University, Zhejiang, China; 4 Department of Preventive Medicine, School of Environmental Science and Public Health, Wenzhou Medical University, Zhejiang, China; 5 Pneumology Department, The First Affiliated Hospital of Wenzhou Medical University, Zhejiang, China; Nippon Medical School Graduate School of Medicine, JAPAN

## Abstract

**Objective:**

The aim of this study was to assess cardiovascular involvement in patients with connective tissue disease (CTD), and determine whether interstitial lung disease (ILD) in these patients is associated with elevated cardiovascular risk.

**Methods:**

This study evaluated a retrospective cohort of 436 CTD patients admitted to a large teaching hospital in Zhejiang province, China, along with an additional 436 participants of an annual community health screening conducted in the physical examination center who served as age- and gender-matched controls. Demographic, clinical, serologic and imaging characteristics, as well as medications used by each participant were recorded. Cardiovascular involvement was defined by uniform criteria. Correlations between clinical/serologic factors and cardiovascular involvement were determined by univariate and multivariate analyses.

**Results:**

CTD patients had a significantly higher cardiovascular involvement rate than controls (64.7% *vs* 23.4%), with higher rates of diabetes, hypertension, and hyperlipidemia, elevated systolic and diastolic pressures, C-reactive protein, total cholesterol, and low-density lipoprotein cholesterol, and lower albumin and high-density lipoprotein cholesterol (all *p* < 0.05). Furthermore, CTP patients with cardiovascular involvement were significantly older, had higher systolic and diastolic pressures, C-reactive protein, glucose, and uric acid, higher rates of diabetes, hypertension, and use of moderate- to high-dose glucocorticoids, and longer disease duration compared to patients without involvement (all *p* < 0.05). Moreover, CTD in patients with cardiovascular involvement was more likely to be complicated by ILD (*p* < 0.01), which manifested as a higher alveolar inflammation score (*p* < 0.05). In the multivariate analysis, cardiovascular involvement in CTD patients was associated with age, systolic pressure, body mass index, uric acid, disease duration > 2 years, use of moderate- to high-dose glucocorticoids, and ILD with a high alveolar inflammation score.

**Conclusion:**

Cardiovascular involvement is increased in CTD patients, and is associated with ILD with a higher alveolar inflammation score. Thus, early-stage echocardiography and CT scans should be used to detect potential cardiovascular complications in these patients.

## Introduction

Connective tissue diseases (CTDs) represent a spectrum of systemic autoimmune diseases characterized by the presence of circulating autoantibodies and significant autoimmune-mediated organ damage. Although new promising therapies are emerging, CTDs are still considered incurable, particularly for patients with various complications. One of the major causes of morbidity and mortality of CTD patients is cardiovascular involvement [1]. The early phases of cardiovascular disease (CVD) are typically asymptomatic, occur at younger ages, and are only characterized by specific risk factors [2, 3]. As a result, CVD is relatively difficult to detect in CTD patients prior to serious, or even fatal, events. Therefore, a regular assessment of CVD risk factors has been recommended in patients presenting with systemic lupus erythematous (SLE), rheumatoid arthritis (RA), and systemic sclerosis (SSc) [4, 5].

Patients with CTDs often exhibit autoimmune damage to the lungs, particularly inflammation and interstitial fibrosis of the lung parenchyma, which is thus described as CTD-associated interstitial lung disease (ILD). CTD-ILD, one of the most common forms of ILD, is often asymptomatic, but can be detected by computed tomography (CT) of the chest and pulmonary function tests. Recent studies of CTD cohorts have shown that the radiographic prevalence of subclinical ILD ranges from 33% to 57% [6–8]. Clinical and serologic data indicate that CTD-ILD is often involved in cases of RA, SSc, polymyositis and dermatomyositis, primary Sjogren’s syndrome, SLE, and mixed and undifferentiated CTD [9].

Accelerated atherosclerosis is considered the primary cause of CVD. Many CTD patients have common risk factors for CVD, including hypertension, diabetes and hypercholesterolemia, in addition to CTD-specific risk factors that may account for elevated cardiovascular morbidity, such as chronic inflammation [10, 11], endothelial dysfunction [12], altered lipid profiles and function [13, 14], oxidative stress, the activity and duration of the autoimmune disease, hypercoagulability, and platelet activation. Moreover, side effects of immunotherapy [15], particularly from glucocorticoids and non-steroid anti-inflammatory drugs, can also contribute to CVD risk. These risk factors also exist in ILD patients [16, 17].

Despite these observations, rheumatologists and respiratory physicians do not typically address the possible cardiovascular risk, which may be elevated by ILD in CTD patients. Thus, there has been very limited research investigating the role of ILD in the progression of cardiovascular involvement in CTD patients. It is therefore necessary to identify the risk factors for CVD in CTD patients in order to improve survival. We conducted a retrospective study to characterize and quantify cardiovascular involvement in a cohort of CTD patients and controls, as well as to investigate the role of ILD for CVD in CTD patients.

## Methods

### Study population

A total of 436 patients with CTDs receiving treatment from the Rheumatology and Pneumology departments of the First Affiliated Hospital of Wenzhou Medical University between January 1, 2008 and December 31, 2013 were enrolled in this study. Another 436 participants from an annual community health screening in the physical examination center of the same hospital were enrolled to serve as an age- and gender-matched control group. The diagnoses of CTDs were determined by the treating rheumatologist, and confirmed by a review of medical records. Patients were diagnosed with SSc, RA, SLE, or primary Sjogren’s syndrome according to American College of Rheumatology criteria [18–21], while the Bohan and Peter criteria were used to diagnose inflammatory myopathies, including polymyositis and dermatomyositis. The diagnosis of mixed CTD was based on clinical features described by Sharp *et al*. [22], and undifferentiated CTD was diagnosed according to the criteria of Mosca *et al*. [9]. Patients with multiple CTD diagnoses or previously diagnosed lung diseases were excluded from the study.

Diagnosis of ILD was determined together by an ILD-expert clinician and a chest radiologist, and was based on serology, clinical signs, and high-resolution (HR) CT of the chest. The cohort included patients with subclinical (changes on HRCT only) and clinical (also with a restrictive pattern on pulmonary function tests or symptoms) ILD diagnoses.

This study was approved by the Ethical Committee of the First Affiliated Hospital of Wenzhou Medical University. Written informed consent was provided by all patients and participants prior to enrollment in the study.

### Clinical data

Clinical signs of CTDs were collected from a review of the patient’s medical records, and data were entered into the study database upon patient enrollment, including clinical presentations, physical and radiologic findings, electrocardiogram (ECG), echocardiograph, and right-heart catheterization data, and laboratory results of serologic tests for antinuclear antibody, baseline immunoglobulin G, C-reactive protein (CRP), albumin, glucose, blood lipid spectrum, and uric acid (UA) levels. Demographic information was also collected, including age at diagnosis, sex, smoking and drinking history, family history of disease, drug use history, and body mass index (BMI).

### HRCT analysis

HRCT of the lungs was performed using 1.2-mm-thick sections to evaluate pulmonary abnormalities. Scans were performed during suspended inspiration, with patients in the supine position. Images were analyzed by a pulmonologist and a radiologist with ILD experience. CT images were assessed for the presence and extent of parenchymal abnormalities, including reticular opacity, honeycombing, ground-glass opacity, emphysema, traction bronchiectasis, and architectural distortion. Previously validated HRCT scores were used [23]. The extent of ground-glass attenuation and honeycombing pattern in each lobe, which represent the alveolar and interstitial fibrosis findings, respectively, were scored on a scale of 0–5 based on the percentage of each lobe involved and the type of involvement. The scores for each lobe from the two readers were averaged for data analyses.

### Cardiovascular evaluation

All patients received ECGs and echocardiograms. A portion of patients received a comprehensive transthoracic echocardiogram when regular echocardiogram results were unsatisfactory. Confirmatory right-heart catheterization was performed in a minority of patients with pulmonary hypertension assessed by echocardiogram. Echocardiography was performed by experienced cardiologists with expertise in CVD; left ventricular-ejection fraction, right-ventricular dimensions, and function were recorded. Systolic pulmonary arterial pressure was estimated by quantifying the maximal velocity of tricuspid regurgitation and adding a two-dimensional estimate of right atrial pressure. Cardiovascular involvement was defined based on the following criteria: 1) arrhythmia (frequent atrial or ventricular premature beats, atrial tachycardia, ventricular tachycardia, atrial fibrillation, and atrioventricular and intraventricular conduction defects); 2) myocardial function limitation (atrial and/or ventricular enlargement, cardiac hypertrophy, ventricular wall dyskinesia, and systolic and/or diastolic dysfunction by ultrasonic cardiogram); 3) pericardial disease (pericardial effusion or thickening in chest HRCT or echocardiography); 4) pulmonary arterial hypertension (mean pulmonary arterial pressure > 25 mmHg at rest by echocardiograph or > 25 mmHg by right-heart catheterization); 5) valvular disease (abnormalities of valve morphology and function); 6) coronary artery calcium (as detected by HRCT); 7) myocardial ischemia (characteristic ECG change was horizontal or down-sloping ST segment depression was > 0.5 mV in three consecutive leads, and signs of myocardial infarction).

### Treatment

Previous and current medications were recorded, including glucocorticoids (dose and duration), disease-modifying antirheumatic drugs (including methotrexate, leflunomide, azathioprine, mycophenolate mofetil, cyclophosphamide, tacrolimus, and antimalarial agents) and cardiovascular medications (i.e., statins, aspirin, beta-blockers, angiotensin-converting enzyme inhibitors, angiotensin receptor blockers, and calcium ion antagonists).

### Statistical analysis

Continuous data are expressed as mean ± standard deviation, and categorical data are presented as counts and percentages. Comparison of continuous variables was performed using the Wilcoxon rank-sum test. Categorical variables were compared using *χ* ^*2*^ or Fisher’s exact tests as appropriate. To examine correlations between risk factors and cardiovascular involvement, confounding factors were identified based on biologic plausibility and literature review. Variables with a *p* ≤ 0.05 in univariate analysis were then included in a multivariate analysis by using reverse-stepwise multiple logistic regression with a threshold for inclusion in the final model of *p* < 0.2. Statistical significance was defined as a *p* < 0.05. All statistical analyses were performed with SPSS software version 17.0 (SPSS Inc., Chicago, IL, USA).

## Results

Demographic and clinical data collected from the study participants are presented in Table 1. CTD patients were more likely than controls to use alcohol and be diabetic or hypertensive (all *p* < 0.05). Furthermore, CTP patients had higher systolic and diastolic pressures, and higher levels of CRP and total and low-density lipoprotein cholesterols, with lower albumin and high-density lipoprotein cholesterol compared to controls (all *p* < 0.05).

**Table 1:** Demographic and clinical characteristics of the study cohort.

Characteristic	Control (*n* = 436)	CTD (*n* = 436)	*p* value
Age, yr	52.21 ± 15.11	52.42 ± 15.28	0.838
Female	350 (80.3)	350 (80.3)	1.000
Smoke exposure	62 (14.3)	48 (11.0)	0.150
Alcohol use	70 (16.1)	48 (11.0)	0.028
Diabetes	34 (7.8)	52 (11.9)	0.042
Hypertension	78 (17.9)	123 (28.2)	< 0.001
Hyperlipidemia	122 (28.0)	210 (48.2)	< 0.001
BMI	22.90 ± 3.41	22.39 ± 3.27	0.024
Systolic pressure, mmHg	110.90 ± 18.16	130.13 ± 22.90	< 0.001
Diastolic pressure, mmHg	72.68 ± 11.87	77.34 ± 13.31	< 0.001
CRP	8.05 ± 8.97	21.67 ± 19.75	< 0.001
Albumin	37.84 ± 4.31	34.89 ± 5.80	< 0.001
Total cholesterol	3.85 ± 0.87	4.86 ± 1.41	< 0.001
HDL-C	1.18 ± 0.35	1.08 ± 0.35	< 0.001
LDL-C	2.38 ± 0.94	3.01 ± 1.10	< 0.001
Triglyceride	1.78 ± 1.14	1.82 ± 1.16	0.602
Glucose	5.26 ± 1.76	5.45 ± 1.80	0.108
Uric acid	295.86 ± 101.87	302.07 ± 109.93	0.387

BMI: body mass index; CRP: C-reactive protein; CTD: connective tissue disease; HDL-C: high-density lipoprotein cholesterol; LDL-C: low-density lipoprotein cholesterol.

Note: Data are presented as mean ± standard deviation or as n (%).

As shown in Table 2, a significantly greater proportion of CTD patients had cardiovascular involvement than controls, including higher rates of arrhythmia, myocardial function limitation, pericardial disease, pulmonary arterial hypertension, valvular disease, and myocardial ischemia (all *p* < 0.001).

**Table 2:** Subtypes of cardiovascular involvement, *n* (%).

Manifestation	Control (*n* = 436)	CTD (*n* = 436)	*p* value
Cardiovascular involvement	102 (23.4)	282 (64.7)	< 0.001
Arrhythmia	44 (10.1)	104 (23.9)	< 0.001
Myocardial function limitation	57 (13.1)	184 (42.4)	< 0.001
Pericardial disease	9 (2.1)	54 (12.4)	< 0.001
Pulmonary arterial hypertension	10 (2.3)	66 (15.1)	< 0.001
Valvular disease	56 (12.8)	110 (25.2)	< 0.001
Coronary artery calcium	29 (6.7)	35 (8.0)	0.442
Myocardial ischemia	35 (8.0)	77 (17.7)	< 0.001

CTD: connective tissue disease.

Comparative analyses revealed that CTD patients with cardiovascular involvement were significantly older, more likely to have a disease duration > 2 years, and exhibit higher BMI, systolic and diastolic pressures, CRP, glucose, and UA (all *p* < 0.05) (Table 3). Furthermore, CTD patients with cardiovascular involvement had higher rates of diabetes, hypertension, ILD, and previous use of moderate- to high-dose glucocorticoids than CTD patients without cardiovascular involvement (all *p* < 0.05). The score of alveolar inflammation, indicating a ground-glass pattern in ILD, was significantly higher in CTD patients with cardiovascular involvement than those without (*p* < 0.05).

**Table 3:** Demographic and clinical characteristics according to cardiovascular involvement.

Characteristic	CTD without cardiovascular involvement (*n* = 154)	CTD with cardiovascular involvement (*n* = 282)	*p* value
Age	46.40 ± 14.73	55.72 ± 14.58	< 0.001
Female	122 (79.2)	228 (80.9)	0.683
Disease duration > 2 yr	28 (18.2)	82 (29.1)	0.012
Smoke exposure	18 (11.7)	30 (10.6)	0.738
Alcohol use	17 (11.0)	31 (11.0)	0.988
Diabetes	9 (5.8)	43 (15.2)	0.004
Hypertension	24 (15.6)	99 (35.1)	< 0.001
Hyperlipidemia	67 (43.5)	143 (50.7)	0.150
ILD	56 (36.4)	164 (58.2)	< 0.001
Alveolar score	0.84 ± 0.80	1.16 ± 0.92	0.021
Interstitial score	0.80 ± 0.75	0.80 ± 0.78	0.972
BMI	21.54 ± 3.23	22.85 ± 3.20	< 0.001
Systolic pressure	122.38 ± 21.59	134.37 ± 22.52	< 0.001
Diastolic pressure	75.44 ± 12.79	78.38 ± 13.50	0.027
CRP	19.07 ± 20.54	23.08 ± 19.20	0.043
Albumin	35.25 ± 6.26	34.69 ± 5.53	0.342
Total cholesterol	4.76 ± 1.50	4.92 ± 1.35	0.251
HDL-C	1.05 ± 0.30	1.10 ± 0.37	0.137
LDL-C	2.95 ± 1.23	3.04 ± 1.03	0.381
Triglyceride	1.73 ± 1.04	1.86 ± 1.22	0.279
Glucose	5.06 ± 1.30	5.66 ± 2.00	< 0.001
Uric acid	281.18 ± 108.37	313.48 ± 109.29	0.003
Immune globulin G	16.86 ± 6.50	16.79 ± 7.27	0.920
Antinuclear antibody positive	113 (73.4)	211 (74.8)	0.741
Glucocorticoid use	130 (84.4)	236 (83.7)	0.843
Moderate to high dose	29 (18.8)	83 (29.4)	0.015
Continuous use for > 1 yr	55 (35.7)	119 (42.2)	0.186
Immunosuppressive agent use	142 (92.2)	244 (86.5)	0.075

BMI: body mass index; CRP: C-reactive protein; CTD: connective tissue disease; HDL-C: high-density lipoprotein cholesterol; ILD: interstitial lung disease; LDL-C: low-density lipoprotein cholesterol.

Notes: Disease duration measured from the day diagnosed; moderate- to high-dose glucocorticoid: > 1 mg/kg/d prednisone; glucocorticoid use at anytime for > 2 wk; data are presented as mean ± standard deviation or as n (%).

The various types of CTD demonstrated similar rates of cardiovascular involvement, all above 50% (Table 4). However, the occurrence of pericardial disease differed among the subtypes (*p* < 0.05), and was most often observed in SLE. Patients with RA had slightly higher rates of myocardial ischemia and coronary artery calcium.

**Table 4:** Classification of connective tissue diseases with cardiovascular involvement, *n* (%).

Manifestation	DM/PM	SSC	RA	PSS	SLE	MCTD	UCTD	*p* value
Cardiovascular involvement	53 (61.6)	48 (68.6)	70 (71.4)	59 (62.1)	30 (54.5)	9 (56.2)	13 (81.2)	0.243
Arrhythmia	19 (22.1)	25 (35.7)	23 (23.5)	18 (18.9)	11 (20.0)	3 (18.8)	5 (31.2)	0.238
Myocardial function limitation	32 (37.2)	33 (47.1)	48 (49.0)	38 (40.0)	20 (36.4)	5 (31.2)	8 (50.0)	0.473
Pericardial disease	8(9.4)	9 (12.9)	12 (12.2)	2(2.1)	15 (27.3)	3 (18.8)	0(0.0)	0.004
Pulmonary arterial hypertension	11 (12.8)	17 (24.3)	14 (14.3)	10 (10.5)	10 (18.2)	2 (12.5)	1 (6.2)	0.059
Valvular disease	18 (20.9)	22 (31.4)	27 (27.6)	25 (26.3)	11 (20.0)	3 (18.8)	4 (25.0)	0.709
Coronary artery calcium	10 (11.6)	4 (5.7)	12 (12.2)	2 (2.1)	5 (9.1)	1 (6.2)	1 (6.2)	0.167
Myocardial ischemia	15 (17.4)	12 (17.1)	25 (25.5)	15 (15.8)	5(9.1)	2 (12.5)	3 (18.8)	0.279

DM/PM: polymyositis and dermatomyositis; MCTD: mixed connective tissue disease; PSS: primary Sjogren’s syndrome; RA: rheumatoid arthritis; SLE: systemic lupus erythematous; SSC: systemic sclerosis; UCTD: undifferentiated connective tissue disease.

Overall, the use of cardiovascular medications in CTD patients with or with out cardiovascular involvement was low. Calcium ion antagonists, angiotensin-converting enzyme inhibitors, and angiotensin receptor blockers were prescribed significantly more often in CTD patients with cardiovascular involvement (*p* < 0.05) (Table 5), which may due to higher hypertension prevalence in this population. No significant difference was found between CTD patients with or without cardiovascular involvement with respect to the use of aspirins, statins, and beta-blockers.

**Table 5:** Cardiovascular medication use at study enrollment, *n* (%).

Medication	Total CTD (*n* = 436)	CTD without cardiovascular involvement (*n* = 154)	CTD with cardiovascular involvement (*n* = 282)	*p* value
Aspirin	187 (42.9)	71 (46.1)	116 (41.1)	0.316
Statins	70 (16.1)	20 (13.0)	50 (17.7)	0.197
Beta blockers	16 (3.6)	4 (2.6)	12 (4.3)	0.379
ACEI/ARB	55 (12.6)	12 (7.8)	43 (15.2)	0.025
Calcium ion antagonist	103 (23.6)	25 (16.2)	78 (27.7)	0.007

ACEI/ARB: angiotensin-converting enzyme inhibitor/angiotensin receptor blocker; CTD: connective tissue disease.

The associations between the clinical and serologic factors in CTD patients with cardiovascular involvement were assessed using univariate analyses (Table 6). Several clinical factors were significantly associated with cardiovascular involvement, including age, disease duration > 2 years, the presence of diabetes, hypertension, and ILD, and elevated BMI, systolic and diastolic pressures, CRP, glucose, UA, and alveolar score, and previous use of high-dose glucocorticoids (all *p* < 0.05).

**Table 6:** Univariate analyses of factors associated with cardiovascular involvement in connective tissue disease.

Variable	Odds ratio	95% confidence interval	*p* value
Age	1.043	(1.029–1.058)	< 0.001
Female	1.107	(0.679–1.807)	0.683
Disease duration > 2 yr	1.845	(1.138–2.992)	0.013
Smoke exposure	0.900	(0.484–1.673)	0.738
Alcohol use	0.995	(0.532–1.863)	0.988
Diabetes	2.899	(1.373–6.121)	0.005
Hypertension	2.930	(1.778–4.828)	< 0.001
Hyperlipidemia	1.336	(0.900–1.983)	0.151
ILD	2.432	(1.623–3.646)	< 0.001
Alveolar score	1.537	(1.061–2.226)	0.023
Interstitial score	0.993	(0.669–1.475)	0.972
BMI	1.141	(1.068–1.217)	< 0.001
Systolic pressure	1.026	(1.016–1.036)	< 0.001
Diastolic pressure	1.017	(1.002–1.033)	0.028
CRP	1.011	(1.000–1.022)	0.045
Albumin	0.984	(0.951–1.018)	0.342
Total cholesterol	1.088	(0.942–1.255)	0.251
HDL-C	1.557	(0.868–2.794)	0.138
LDL-C	1.085	(0.904–1.301)	0.381
Triglyceride	1.108	(0.919–1.337)	0.282
Glucose	1.251	(1.094–1.432)	0.001
Uric acid	1.003	(1.001–1.005)	0.004
Immunoglobulin G	0.999	(0.971–1.027)	0.920
Antinuclear antibody positive	1.078	(0.690–1.686)	0.741
Glucocorticoid use	0.947	(0.553–1.622)	0.843
Moderate to high dose	1.798	(1.114–2.900)	0.016
Continuous use > 1 yr	1.314	(0.876–1.971)	0.187
Immunosuppressive agents use	0.542	(0.275–1.072)	0.079

BMI: body mass index; CRP: C-reactive protein; HDL-C: high-density lipoprotein cholesterol; ILD: interstitial lung disease; LDL-C: low-density lipoprotein cholesterol.

Notes: Disease duration measured from the day diagnosed; moderate- to high-dose glucocorticoid: > 1 mg/kg/d prednisone; glucocorticoid use at anytime for > 2 wk.

To avoid interference from risk factors, clinical factors associated with cardiovascular involvement were further analyzed by multivariate analyses controlling for age, disease duration, the presence of diabetes, hypertension, ILD, BMI, systolic and diastolic pressures, CRP, glucose, UA, and use of high-dose glucocorticoids and cardiovascular drugs (Table 7). Age, disease duration > 2 years, presence of ILD, BMI, systolic pressure, UA, alveolar score, and use of high-dose glucocorticoids were all significantly associated with cardiovascular involvement in CTD patients (all *p* < 0.05).

**Table 7:** Multivariate analyses of predictors of cardiovascular involvement in connective tissue disease.

Variable	Odds ratio	95% confidence interval	*p* value
Age	1.027	(1.010–1.044)	0.001
Disease duration > 2 yr	1.794	(1.044–3.086)	0.035
Diabetes	1.401	(0.556–3.528)	0.474
Hypertension	0.923	(0.448–1.901)	0.828
ILD	2.022	(1.251–3.268)	0.004
Alveolar score	1.551	(1.005–2.395)	0.048
Interstitial score	1.024	(0.643–1.631)	0.920
BMI	1.112	(1.035–1.196)	0.004
Systolic pressure	1.018	(1.002–1.034)	0.031
Diastolic pressure	0.995	(0.974–1.017)	0.117
CRP	1.004	(0.992–1.017)	0.483
Glucose	1.057	(0.899–1.242)	0.503
Uric acid	1.003	(1.001–1.005)	0.010
Use of moderate- to high-doseglucocorticoids	1.859	(1.094–3.157)	0.022

BMI: body mass index; CRP: C-reactive protein; ILD: interstitial lung disease.

Notes: Disease duration measured from the day diagnosed; moderate- to high-dose glucocorticoid: > 1 mg/kg/d prednisone.

## Discussion

Cardiovascular involvement in CTDs can be asymptomatic, particularly in its early phases when it manifests as subclinical microcirculation abnormalities. Here we used a number of reliable and noninvasive methods, such as echocardiography and HRCT, to detect preclinical cardiovascular damage, aside from the golden standard of coronary angiography. Echocardiography appears to be the most suitable cardiovascular assessment method in cases of systemic autoimmune diseases [1]. In addition, HRCT is capable of detecting small amounts of calcium in coronary arteries, and the coronary calcium scores obtained by this method can predict cardiac events such as acute myocardial infarction and cardiac death, which are highly associated with the magnitude of plaque burden [24]. Thus, HRCT can also provide a useful tool for stratifying the cardiovascular risk for patients with autoimmune disease.

ILD is a common complication of CTD, and is associated with a poor prognosis [25]. In our retrospective study, 64.7% of all CTD patients exhibited some form of cardiovascular involvement, with much higher prevalence than age- and gender-matched controls. Our findings are consistent with reports of cardiovascular complications in RA, SLE, and SSc patients [1, 2, 26–28]. In addition to the additional risk factors for CVD, such as age, hypertension, BMI, systolic pressure, CRP, UA, we found that ILD was also a specific risk factor for CVD among CTD patients. Whereas the traditional cardiovascular risk factors in CTDs are well understood, the additional risks of ILD are often underestimated due to the masking of cardiovascular symptoms.

ILD in this study was mainly diagnosed by chest HRCT, which can be used to distinguish different subgroups, such as usual and nonspecific interstitial pneumonias, in the absence of pathologic material. However, reaching a consensus on HRCT categories may sometimes be problematic. The agreement on diagnoses of definite or probable usual and nonspecific interstitial pneumonias between two experienced thoracic radiologists was reported to be 35% [29]. Therefore, we used previously validated HRCT scores reporting interstitial fibrosis and alveolar involvement to assess the state of ILD by HRCT [23]. Ground-glass opacity was frequently observed, as previously reported in CTD-ILD, and frequently corresponded to a histopathologically defined pattern of nonspecific interstitial pneumonia [30]. The results show that alveolar findings are associated with cardiovascular involvement, which may be explained by inflammatory injury, endothelial dysfunction [12], oxidative stress, and platelet activation in the acute phase of ILD. Further studies are needed to determine how alveolar inflammation may act to influence cardiovascular performance with distinct presentations.

The clinical manifestations of cardiovascular involvement include rhythm and conduction disturbances, coronary artery calcium, myocardial dysfunction, valvular diseases, pulmonary hypertension, and ischemic heart disease [26, 31]. We found that CTD patients had much higher rates than controls of arrhythmia, myocardial function limitation, pericardial disease, pulmonary arterial hypertension, valvular disease, and myocardial ischemia, suggesting a potential link between CTD and some forms of CVD, consistent with their similar pathologic processes [17, 32]. As CTDs are a spectrum of different diseases, their manifestations of cardiovascular involvement are diverse. Importantly, RA patients were more likely to exhibit myocardial ischemia and coronary artery calcium, which could be easily overlooked in patients with arthritis.

In our study, we also found the present cardioprotective therapy is inadequate despite the high rate of cardiovascular involvement in CTD patients, which reflects the pre-existing perception among our physicians. However, Mäki-Petäjä *et al*. [33] demonstrated the efficacy of ezetimibe and simvastatin in reducing disease activity, systemic inflammation, and aortic stiffness in RA patients, while improving endothelial function. Furthermore, aspirin has been used to reduce thrombotic events for autoimmune diseases associated with hypercoagulable states (e.g., anti-phospholipid syndrome) [34]. For example, Haque *et al*. [35] suggested that aspirin should be administered in select SLE patients to help control cholesterol levels and blood pressure. The low rate of cardiovascular therapy reaffirms previous observations of under-treatment in CTD patients, and represents a major modifiable cardiovascular risk factor, which partly contributes to the high rates of cardiovascular involvement.

Although optimal treatments for CTD patients remain elusive, corticosteroids and immunosuppressive drugs are the most frequently recommended [36]. Corticosteroids can disturb carbohydrate and triglyceride metabolism and aggravate classic risk factors such as hypertension, hyperglycemia, and hyperlipidemia. However, corticosteroids can also reduce nontraditional risk factors such as inflammation [37]. Therefore, it is difficult to determine whether to suspend corticosteroid use in CTD patients when considering cardiovascular protection. In our study, although general or continuous use of glucocorticoids over one year was not associated with cardiovascular involvement in CTD patients, previous use of moderate- to high-dose glucocorticoids was. This suggests that corticosteroid therapy should be prudently and accurately initiated according to clinical need, taking the dose-related potential for cardiovascular risk into account. Different from corticosteroids, immunosuppressive therapy does not substantially affect cardiovascular risk. However, previous evidence indicated that antirheumatic drugs can inhibit atherosclerosis by reducing inflammation and disease activity, while also reversing endothelial dysfunction [38]. Thus, whether immunosuppressive therapies have beneficial effects on cardiovascular damage among patients with CTD still needs further study.

One limitation of our study is its retrospective design, which is subject to recall and selection biases, thus impacting the uniformity of the collected data. In addition, we were unable to draw any conclusions regarding mixed and undifferentiated CTD due to our limited sample size. Moreover, our results reflect the patients and practices of a single tertiary care center, and may not be applicable to other medical institutions. Finally, we did not estimate the severity of CTDs, which could be influenced by cardiovascular involvement, because of the incommensurability of scoring systems for each CTD. Thus, future larger prospective studies are necessary to verify our observations and develop a sound therapeutic recommendation for these patients.

In patients with CTDs, cardiovascular complications primarily present with subclinical features, however these complications are one of the major causes of CTD morbidity and mortality. Thus, it is essential to detect cardiovascular involvement in CTD patients at an early stage. We demonstrate that, apart from risk factors such as age, systolic pressure, BMI, UA, disease duration, and use of moderate- to high-dose glucocorticoids, ILD with higher alveolar inflammation scores are associated with cardiovascular complications in CTD patients. Our data suggest that CTD patients, particularly those with ILD, should undergo cardiovascular monitoring and cardioprotective therapy in order to improve patient outcome.

## Supporting Information

S1 Dataset Demographic, clinical, serologic and imageological characteristics and medications record of the Study Cohort. (XLS)
